# The Most Marvelous Flower in the World--Your Baby!

**Published:** 1914-02

**Authors:** B. W. Hamilton

**Affiliations:** Delineator


### The Most Marvelous Flower in the World--Your Baby!

BY DR. B. W. HAMILTON, IN DELINEATOR.

It's rather a difficult business, that of being a baby. If we can help along the business, we ought to, and I believe that a big percentage of babies die every year because they do not have enough sunshine and fresh air. We are beginning to realize that our babies are like flowers; sunshine keeps them from illness; it cures them, too.

If it is possible, let your baby live in a south room where there is plenty of light, air and sunshine. If such a room is not available for day and night use, trundle the baby in his carriage into your sunniest room in the daytime and put him to sleep in your airiest room at night.

I wish that every baby might have its own out-of-door sleeping-room. Such a room would cut doctor's bills in half and give our children the robust health in the future that we ought to build for them in babyhood.

*Fit Up Your Own Outdoor Nursery.*—An outdoor nursery does not mean a big money outlay at the beginning. It doesn't mean that you will have to build on a sleeping-porch to your house with the arrival of your first baby. It means just making the best use of that sunny back porch of yours, giving over one end of it and furnishing this airy corner for the accommodation of your baby.

A porch-end large enough to conveniently hold the baby's carriage and a table with necessary appliances is all that is necessary. Your first care will be to ward against drafts. Baby can stand a great deal of fresh air blowing in from overhead, but we must keep his body warm.

To provide for keeping the baby out in all kinds of weather, it will be a good plan to glass in one end of the porch. The window frames should be put in in such a way that they will either swing out and in or raise and lower easily, because they are intended to be storm-guards. Never must they shut out fresh, life-giving air. A heavy curtain fitted with rings and hung on a rod will be a good storm and wind-guard, if the glass window is not available.

Will the baby suffer if he naps and plays and naps again here in the open?

Not a bit.

A heavy blanket should be folded so as to result in several thicknesses and laid in the bottom of the baby's carriage beneath his pillows. A hot-water bag or a heated soapstone will keep his feet comfortable. He should have specially heavy underflannels, a flannelet slip, leggings and sweater, in addition to his coat, for this outdoor living. Wrapped in blankets, he will be completely warm, comfortable and happy.

One mother complained to me: "I want my six-months-old baby to take his nap out-of-doors, Dr. Hamilton, but he cries so that I have to give it up. What shall I do?"

I found upon making further inquiry that this mother had not changed her baby's position in his carriage often enough. He couldn't move about comfortably. He cried because his muscles were cramped from lying in one position. This difficulty overcome, the crying ceased and the baby began to show the benefits of his outdoor naps.

The furnishings of your nursery should be of the simplest—an enameled bed, and all draperies and rugs of light, washable material. The floor should not be carpeted. The floor should be wiped daily with a damp cloth, *never* dusted. Thoroughly air baby's room for at least an hour morning and evening. The best time for this is after his morning bath when he is out-of-doors, and before bedtime.

*The Question of Ventilation.*—Nurseries are almost invariably overheated. How shall we get proper ventilation without drafts? Open fires which, unfortunately, we all haven't, are the best means of ventilating and heating. The most simple ventilation system is a window-board six inches wide and long enough to exactly fit the window's width. This board is placed beneath the raised lower sash, insuring free passage of air upward into the room between the sashes. The proper average temperature of the room in the daytime should be 68 degrees Fahrenheit. Always keep a window open at night, unless the temperature out-of-doors is below 35 degrees.

Do not have oil or gas stoves in the baby's room. If you have steam heat or hot air, place a pan of water beneath the radiator or in the room. This is to prevent excessive drying of the air, one of the most frequent causes of head colds in very young children.

A gas-jet allowed to burn in the sleeping-room at night makes your baby starve for oxygen. When you have no electric light, use wax-candle night-lights.

In flytime the windows should be fitted with screens to keep out flies and mosquitoes. The latter often poison a baby and sometimes transmit malaria.

The fly is both filthy and dangerous because he carries gems on his feet and may light on the nipple of the baby's bottle or on his food.

I wish that I might go on further this month, but our letterbox needs opening. If I don't reach *your* baby this month, I shall soon.

